# B-Myb Induces *APOBEC3B* Expression Leading to Somatic Mutation in Multiple Cancers

**DOI:** 10.1038/srep44089

**Published:** 2017-03-09

**Authors:** Wen-Cheng Chou, Wei-Ting Chen, Chia-Ni Hsiung, Ling-Yueh Hu, Jyh-Cherng Yu, Huan-Ming Hsu, Chen-Yang Shen

**Affiliations:** 1 Institute of Biomedical Sciences, Academia Sinica, Taipei, Taiwan; 2 Department of Surgery, Tri-Service General Hospital, Taipei, Taiwan; 3 Taiwan Biobank, Academia Sinica, Taipei, Taiwan; 4 College of Public Health, China Medical University, Taichung, Taiwan

## Abstract

The key signature of cancer genomes is the accumulation of DNA mutations, the most abundant of which is the cytosine-to-thymine (C-to-T) transition that results from cytosine deamination. Analysis of The Cancer Genome Atlas (TCGA) database has demonstrated that this transition is caused mainly by upregulation of the cytosine deaminase *APOBEC3B (A3B*), but the mechanism has not been completely characterized. We found that B-Myb (encoded by *MYBL2*) binds the *A3B* promoter, causing transactivation, and this is responsible for the C-to-T transitions and DNA hypermutation in breast cancer cells. Analysis of TCGA database yielded similar results, supporting that *MYBL2* and *A3B* are upregulated and putatively promote C-to-T transitions in multiple cancer types. Moreover, blockade of EGF receptor with afatinib attenuated B-Myb–A3B signaling, suggesting a clinically relevant means of suppressing mutagenesis. Our results suggest that B-Myb–A3B contributes to DNA damage and could be targeted by inhibiting EGF receptor.

Accumulation of somatic mutations is a hallmark of nearly every cancer^1^,^2^,^3^,^4^, and emerging technologies for high-throughput sequencing have enabled the identification of numerous somatic mutations in tumor samples^5^,^6^,^7^. Analysis of mutational signatures has identified C-to-T and G-to-A transitions as the most abundant mutation types among the 12 types of base substitutions in human cancers, and both of these transitions are thought to be predominantly catalyzed by cytosine deaminases^4^,^8^,^9^,^10^. In humans, 11 genes are known to contain conserved DNA cytosine deaminase domains, including *AID, APOBEC1, APOBEC2*, the *APOBEC3* gene families *A, B, C, D, F, G, H*, and *APOBEC4*^1^. Among these, *APOBEC3*s encode host defense factors that deaminate deoxycytidine residues on minus-strand DNA of retroviruses such as HIV-1^11^. In addition to deaminating viral genome, evidence also suggests that both APOBEC3A (A3A) and APOBEC3B (A3B) can catalyze C-to-T transitions in DNA, and these enzymes are strongly associated with cytosine-derived somatic mutations in cancer genomes, but only endogenous A3B is frequently upregulated in certain cancer types such as breast, ovary, head/neck, esophagus, among others^8^,^12^,^13^. Elevated *A3B* expression was recently reported to correlate with poor prognosis in breast, gastric, kidney, and lung cancers, implying that A3B-mediated mutagenesis is important for tumorigenesis in humans^14^,^15^,^16^,^17^.

Given that chromosomal rearrangements and copy number variations are infrequent in the region flanking the *A3B* locus as well as a lack of promoter demethylation at adjacent CpG islands, and thus the observed upregulation of *A3B* in breast cancers is presumably due to upstream signal transduction^12^. One such possible signal transduction mechanism involves infection with human papillomavirus (HPV), i.e., the HPV strain HPV16 or HPV18 induces *A3B* expression in cultured cells of breast and head/neck cancers, and the virus-encoded protein E6 directly binds the proximal *A3B* promoter and triggers transcription; however, the prevalence of HPV involvement in cancers is not fully known^18^,^19^. Subsequent work also demonstrated the involvement of the zinc-finger protein ZNF384 in HPV-associated human cancers, although ZNF384 is not required for basal *A3B* expression^20^. Two recent studies reported that phorbol-myristic acid induces *A3B*, and the researchers subsequently found that protein kinase C (PKC)-mediated NF-κB signaling is responsible for *A3B* upregulation^21^,^22^. Although inhibition of PKC markedly represses *A3B* expression in various cancer cell types, it has not been confirmed whether *A3B* upregulation is associated with dysregulated PKC and NF-κB activities in those tumors. Considering the existing literature that has identified transcription factors regulating APOBEC3B expression, but none of them were confirmed associates with A3B upregulation and somatic mutations in clinical tumors. Hence, we scanned the *A3B* promoter–proximal region and identified myb-related protein B (B-Myb) as a regulator of *A3B* expression in breast cancer cells. Indeed, we found B-Myb is a ubiquitous transcription factor that is upregulated in multiple cancer types. We also found that B-Myb overexpression caused DNA editing at sites preferentially targeted by A3B, suggesting that B-Myb is associated with C-to-T mutations in tumor samples. Moreover, we found that signaling mediated by the epidermal growth factor receptor (EGFR) participates in *MYBL2* and *A3B* expression in cancer cells. Together with the aforementioned findings, we conclude that B-Myb–A3B signaling is responsible for cancer mutagenesis and thus could be targeted via EGFR inhibition.

## Results

### Identification of Putative DNA Binding Elements in the *A3B* Core Promoter

**Figure 1:**
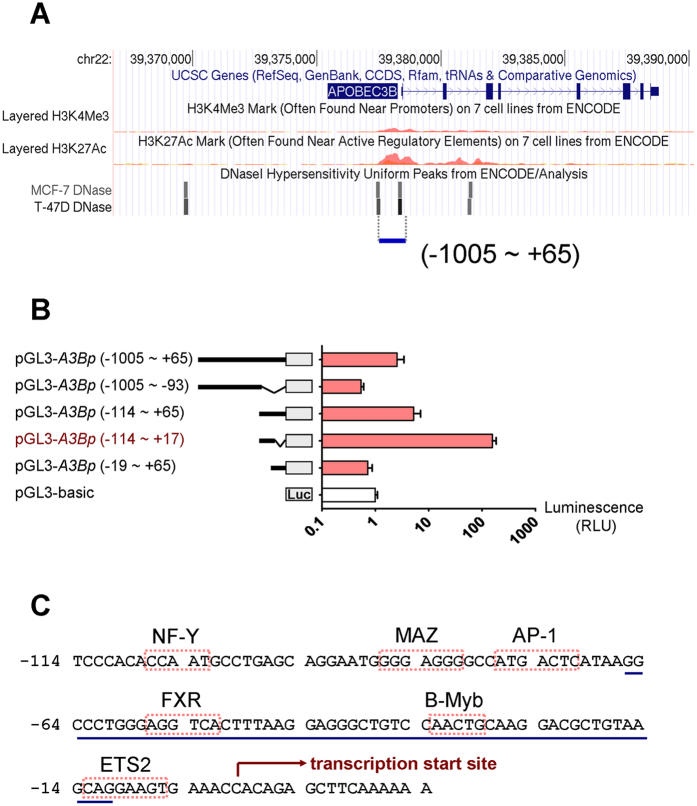
Identification of candidate transcription factor motifs within the *A3B* core promoter region. (**A**) Genomic context around the *A3B* sequence as displayed by the UCSC ENCODE browser. (**B**) pGL3 plasmid containing different portions of the *A3B* promoter or empty plasmid (pGL3-basic) was transfected into MCF7 cells for 48 h. Luciferase expression was measured and normalized to that measured with the pGL3-basic group. (**C**) Prediction of probable transcription factor binding motifs within the *A3B* core promoter region (−114 ~ +17). Six elements and corresponding transcription factors and putative TSS are indicated.

To determine probable factors responsible for *A3B* expression in breast cancer cells, initially we searched the UCSC ENCODE browser and retrieved proximal sequence upstream of the *A3B* transcription start site (TSS) as the candidate *A3B* promoter. The ~1.1-kb promoter contains two DNase I–hypersensitive sites and is enriched with histone H3 lysine 27 acetylation sites adjacent to the TSS (Fig. 1A). This sequence was then divided into segments of differing length and sequentially inserted into vector pGL3 for luciferase reporter analysis (Fig. 1B). To identify the active promoter region, human breast cancer cells (MCF7) were transfected with these plasmids. Compared with empty pGL3, luciferase activity from the region (−114 ~ +17) was induced by >150-fold; in contrast, the region (−19 ~ +65) showed 30% reduction, suggesting that the minimal region (−114 ~ −19) might contain essential elements responsible for basal expression of *A3B* (Fig. 1B). Next, we analyzed our defined region (−114 ~ +17) and found at least six putative consensus binding elements as predicted with the MAPPER and MotifMap search engines (Fig. 1C). Hence, six genes including *ETS2, NR1H4* (FXR protein), *JUN* (AP-1 protein), *MAZ, MYBL2*, and *NFYA*, corresponding to those elements, were considered as putative transcription factor and inserted individually into mammalian expression vectors.

### B-Myb Is a Transcription Factor Responsible for *A3B* Overexpression in Breast Cancer

**Figure 2:**
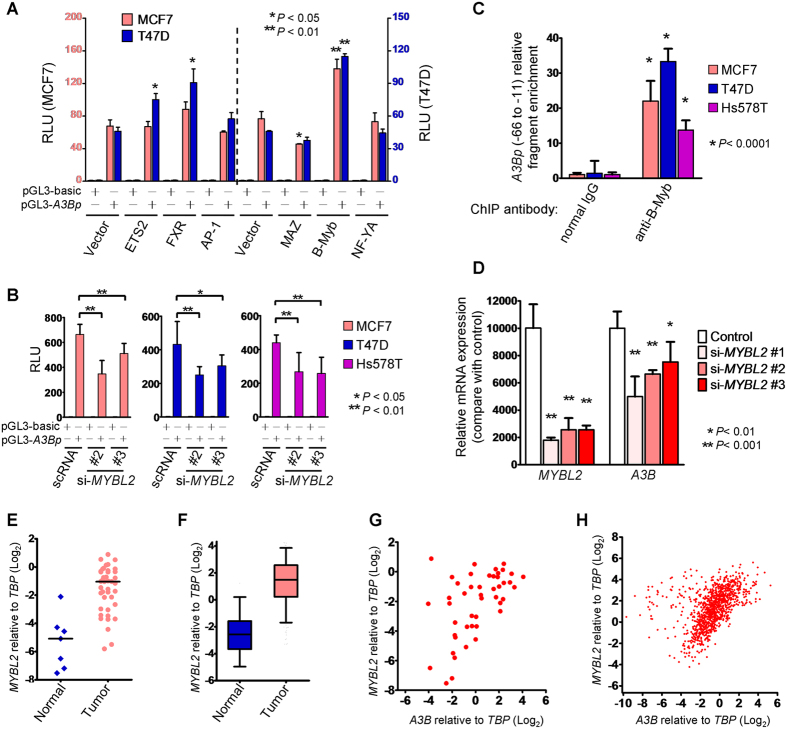
B-Myb is the transcription factor responsible for *A3B* upregulation in breast cancer. (**A**) pGL3 containing the *A3B* promoter (*A3Bp*, −114 ~ +17) or empty plasmid (pGL3-basic) was co-transfected with the indicated Flag-tagged–expressing pcDNA3 vectors into MCF7 or T47D cells. The histogram reports relative luciferase units (RLU) referred to as promoter activity, which was normalized to expression of pGL3-basic control (pGL3-basic and pcDNA3). Asterisks indicate a significant change in promoter activity compared with pGL3-*A3Bp* control (pGL3-*A3Bp* and pcDNA3) that was mediated by the various transcription factors indicated on the bottom axis. (**B**) pGL3-basic or pGL3 containing *A3Bp* was co-transfected with siRNAs targeting *MYBL2* mRNA into breast cancer cells. scRNA: scrambled/control siRNA. Luciferase activity was measured and normalized as in (**A**). (**C**) Histogram showing DNA fragment enrichment after chromatin immunoprecipitation with normal rabbit IgG (control) or anti-B-Myb. Significant enrichment was compared using the Student’s *t*-test. (**D**) Histogram showing relative mRNA expression and 95% confidence interval for each of *MYBL2* and *A3B*. Values were normalized to *TBP*. MCF7 cells were transfected with control siRNA or siRNA targeting *MYBL2* for 48 h. After RNA extraction, *MYBL2* and *A3B* mRNA levels were measured by quantitative RT-PCR. Asterisks indicate that *MYBL2* or *A3B* expression in those treatments differed significantly from the control. (**E**) *MYBL2* mRNA levels from the cDNA array panel including 7 normal breast samples and 39 breast tumor samples were measured using quantitative RT-PCR and normalized to *TBP* levels. Significant *MYBL2* upregulation in tumors relative to normal tissues was determined with the Mann–Whitney *U*-test (*P* = 0.0003). (**F**) Histogram showing the median value and 5th–95th percentile range of *MYBL2* mRNA levels queried from TCGA breast cancer dataset. Upregulation of *MYBL2* mRNA in tumors relative to normal tissues was determined with the Mann–Whitney *U*-test (*P* < 0.0001). (**G**,**H**) Scatter plots showing normalized *A3B (x* axis) and *MYBL2 (y* axis) expression as log-transformed values. Each data point represents one sample that was obtained from (**G**) a cDNA array panel or (**H**) TCGA breast cancer dataset. Correlations between *A3B* and *MYBL2* expression were determined using Spearman’s rank test.

Based on our results above concerning the *A3B* basal promoter region having the strongest luciferase reporter activity, i.e., the region −114 ~ +17, we transfected vector pGL3 containing this region (or empty pGL3) into MCF7 cells and another human breast cancer line (T47D) and measured luciferase activity via co-transfection with empty vector or plasmids expressing the putative transcription factor. Among these transcription factor genes, we found that only MAZ inhibited *A3B* reporter activity, but this difference was significant only in MCF7 cells (Fig. 2A). Interestingly, only B-Myb significantly enhanced *A3B* reporter activity in both cell lines (Fig. 2A). In contrast, small interfering RNA (siRNA)-mediated knockdown of *MYBL2* downregulated reporter activity in MCF7 and T47D cells as well as the estrogen receptor–negative breast cancer line Hs578T, revealing that B-Myb might modulate *A3B* promoter activity in breast cancer cells (Fig. 2B). We then performed chromatin immunoprecipitation using an antibody specific for B-Myb and confirmed that B-Myb indeed localized to the minimal promoter region (−66 ~ −11; Fig. 2C). Using MCF7 cells, we also knocked down *MYBL2* using different siRNA pairs and found that *A3B* mRNA level also decreased (Fig. 2D), which was consistent with the results presented in Fig. 2B. These results indicated that B-Myb is a novel upstream regulator *A3B* in breast cancer cells.

B-Myb, and its homologs c-Myb (encoded by *MYB*) and A-Myb (*MYBL1*), comprise the MYB family of transcription factors that bind the consensus MYB binding site in gene promoters; expression of *MYB* and *MYBL1* is tissue specific in mammals, whereas *MYBL2* is ubiquitously expressed^23^. Because *MYBL2* expression correlates with poor prognosis of breast cancer patients, it has been utilized as an independent biomarker to predict breast cancer recurrence^24^. We therefore quantified mRNAs from 39 tumors and 7 normal samples and found that *MYBL2* was markedly upregulated in tumors (Fig. 2E). This upregulation of *MYBL2* in breast tumorigenesis was substantiated by an analysis of mRNA expression data (RNAseqV2) for 1090 breast tumor samples and 112 normal samples as retrieved from The Cancer Genome Atlas (TCGA) project (Fig. 2F). We then assessed *MYBL2* and *A3B* expression in the aforementioned 39 tumor/7 normal samples and found a correlation between their expression (Spearman’s r = 0.5086, *P* = 0.0003; Fig. 2G). Moreover, the expression of these two genes was also remarkably correlated in an analysis of TCGA breast cancer dataset (Spearman’s r = 0.569, *P* < 0.0001; Fig. 2H). Thus, our results, including cell-based and clinical analyses, supported the idea that *MYBL2* promotes *A3B* induction in human breast tumors.

### A3B and B-Myb Enhance DNA Editing and Somatic Mutation in Breast Cancer

**Figure 3:**
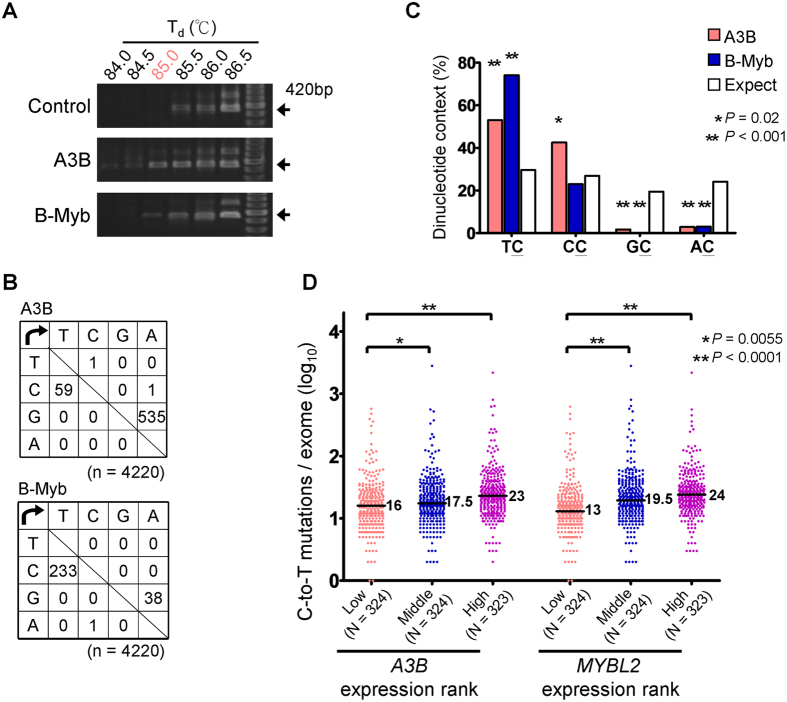
*A3B* and *MYBL2* upregulation lead to somatic mutation in breast cancer. (**A**) Agarose gel electrophoresis shows 3D-PCR products amplified from foreign DNA (*Luc*+) at the indicated denaturation temperatures (T_d_). MCF7 cells were transfected with *A3B, MYBL2*, or empty vector together with pGL3 and pcDNA3-*UGI*. Total DNA was recovered for 3D-PCR analysis after transfection for 48 h. Each arrow denotes 420 bp. (**B**) Mutation metrics for *Luc*+ sequence derived from 3D-PCR amplicons at T_d_ = 85 °C. Ten clones were directly sequenced for each sample, and the total number of bases sequenced (n) is indicated. (**C**) Dinucleotide context analysis for the rate of C-to-T or G-to-A mutation at the indicated sequence for the *Luc*+ sequence shown in (**B**). Asterisks indicate statistical significance compared with the expected mutation rate as assessed with the χ^2^ test. (**D**) Each data point represents the C-to-T mutation count for one sample in TCGA breast cancer dataset. Data were grouped based on low, middle, and high third ranks for *A3B* or *MYBL2* expression. Horizontal bars indicate median values, and statistical significance between two groups was determined with the Mann–Whitney *U*-test.

Because A3B was shown to be responsible for conferring C-to-T transitions via deamination of human DNA^25^, A3B is a potential hazard factor for inducing somatic mutations and genomic instability. Hence, B-Myb, via its regulation of A3B expression, could be a factor that contributes to somatic mutations in breast tumors. To test this possibility, we initially tested the mutagenic capacity of B-Myb using differential DNA denaturation PCR (3D-PCR). 3D-PCR is based on the fact that DNA with a relatively greater A/T content—e.g., upon C-to-T editing—can be amplified at a lower denaturation temperature (T_d_) compared with unmodified sequences. We prepared pGL3 encoding luciferase (*Luc*+) to serve as the DNA to be edited, and then we calculated the number of base substitutions after recovering the plasmid from MCF7 cells. The *Luc*+ sequence could be amplified at a relatively lower T_d_ of 84 °C or 85 °C for the A3B- or B-Myb–expressing group, respectively, in contrast to 85.5 °C for the control group (Fig. 3A). This result indicated the amplicons had different nucleotide compositions at a T_d_ < 85.5 °C, and therefore we cloned the amplicon acquired at T_d_ = 85 °C and performed sequencing. The mutation matrices revealed a high level of C-to-T or G-to-A mutations, and the frequency of mutations was greater in the A3B-expressing group compared with the B-Myb–expressing group (Fig. 3B). We further analyzed the dinucleotide context of C-to-T or G-to-A mutations in the sequenced region and found that both A3B- and B-Myb–expressing cells displayed a strong bias toward deamination of C residues flanked by 5′ T residues, which was recognized as A3B preferred C residues (Fig. 3C). This finding was confirmed by comparing RNA and exome sequence results from TCGA breast cancer dataset, which revealed a significant association between *MYBL2* and *A3B* expression and somatic mutations in that, once mutations occurred in tumors, there were twice as many C-to-T as well as total mutations per exome in higher *MYBL2*–expressing cells compared with lower *MYBL2*–expressing cells (Fig. 3D and Supplementary Fig. S1). Hence we concluded that B-Myb may transactivate *A3B*, leading to somatic mutation in breast tumors.

### rs619289 Is an eQTL for *MYBL2* Expression Associated with Breast Cancer Progression

**Figure 4:**
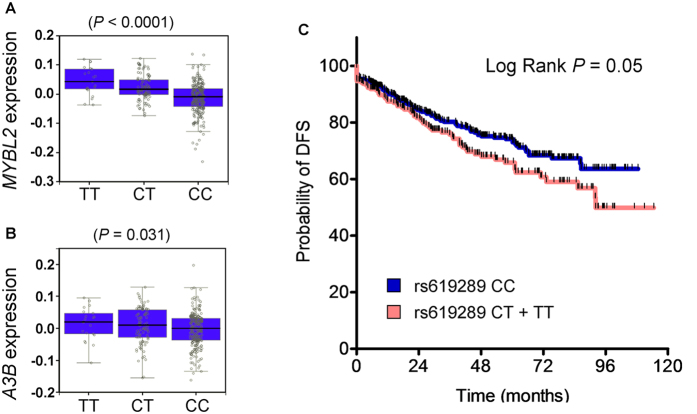
An eQTL polymorphism of *MYBL2* is associated with poor prognosis of breast cancer patients. (**A**,**B**) The distribution of (**A**) *MYBL2* and (**B**) *A3B* expression sorted by genotype of rs619289 in whole-blood samples was queried from the GTEx Portal. The number of samples harboring TT, CT, and CC alleles was 21, 92, and 225, respectively. (**C**) Disease-free survival (DFS) of breast cancer patients (stage ≥II) harboring wild-type (CC, 487 samples) or variant-type (CT + TT, 230 samples) rs619289. The data were adjusted for factors associated with breast cancer progression, e.g., patient age, tumor stage, and hormone therapy status.

Our finding that somatic mutation is regulated by the B-Myb–A3B pathway in breast cancer led us to address the relevance of this pathway to clinical outcome. If overexpression of *MYBL2*, as well as *A3B*, is associated with breast cancer progression, polymorphisms in the expression quantitative trait loci (eQTL) that are associated with *MYBL2* mRNA expression would be expected to be significantly associated with disease outcome. Based on a literature survey, a single nucleotide polymorphism (SNP), rs619289, which is located in upstream of *MYBL2*, was investigated^26^. To verify the role of this SNP in cancer risk, we performed genotype and expression correlation analysis by retrieving the genotype of rs619289 and *MYBL2* expression from the GTEx portal^27^. We found that the T allele of rs619289 from blood samples correlated with increased expression of *MYBL2* and *A3B* but not with expression of the other adjacent *APOBEC3* genes (Fig. 4A,B; Supplementary Fig. S2). Because the rs619289 genotype impacts *MYBL2* or *A3B* expression, we presumed that patients carrying the T allele would have greater mutation rates and poorer cancer outcomes. Consistent with our speculation, patients with invasive (stage II or higher) breast cancer who carried the T allele had a poorer disease-free survival (Fig. 4C). The Cox proportion hazard model also showed that the T allele was significantly associated with disease-free survival of the same patients (Supplementary Table S1). This result implies that rs619289 is an independent risk factor for breast cancer progression when adjusted for the effects of other risk factors such as cancer stage and the history of hormone therapy.

### *MYBL2* and *A3B* Are Upregulated and Correlate with Somatic Mutation Load in Multiple Cancers

**Figure 5:**
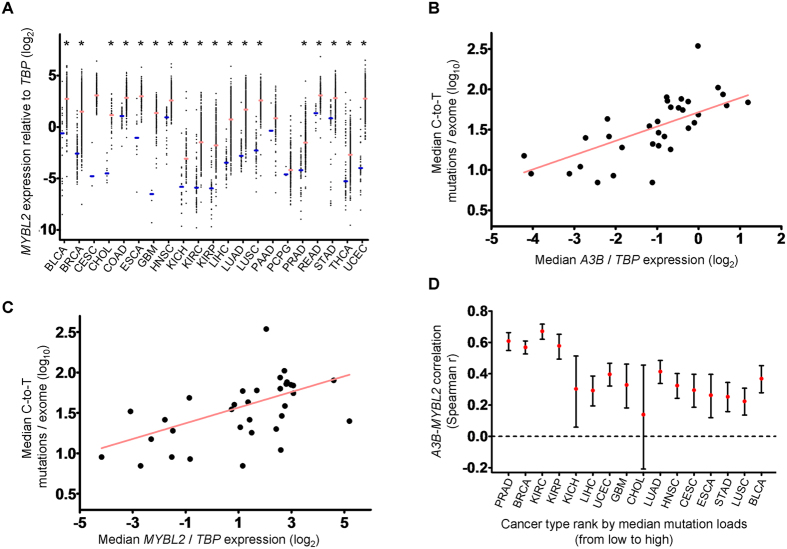
Upregulation of *MYBL2* is evident in multiple cancer types, and its expression correlates with the median level of somatic mutation load. (**A**) Each data point represents relative *MYBL2* expression of one normal or tumor sample, as queried from TCGA RNA sequence database. The *x* axis denotes a total of 21 cancer types, and blue and pink horizontal bars indicate the median *MYBL2* expression (relative to that of *TBP*) for each normal and tumor cancer type, respectively. Asterisks indicate statistically significant *MYBL2* upregulation in the tumor type relative to the corresponding normal tissue. (**B**,**C**) Correlation between C-to-T mutation load (*y* axis) against (**B**) *A3B* or (**C**) *MYBL2* mRNA level relative to *TBP* mRNA (*x* axis). Each data point represents both median values for one cancer type, and the line is the best fit for visualization. The correlation between them was calculated using Spearman’s correlation test. See also Supplementary Tables S4 and S6. (**D**) Each data point and 95% confidence interval represents the correlation status (Spearman’s r, *y* axis) between *A3B* and *MYBL2* mRNA levels for one TCGA cancer type. A total of 16 TCGA cancer types were included in the *x* axis and ranked according to somatic mutation load. See also Supplementary Table S2.

Because *A3B* expression is markedly upregulated and variations in its expression contribute to the different median mutation loads observed between multiple cancers (Supplementary Fig. S3)^8^, we next examined whether this correlation is also valid when *MYBL2* is substituted for *A3B*. To test this, we analyzed transcriptomic and exomic data from TCGA dataset stratified cancer types. Compared with the respective normal control, cancer types (18 of 21 cancers) showed that *MYBL2* expression was significantly upregulated (*P* < 0.0001), implying its common role in cancers of almost every organ (Fig. 5A). Hence, we investigated whether *MYBL2* expression correlates with mutation load in cancers other than breast cancer using 33 cancer types and mutation load data from TCGA. The median values for mutation load and *MYBL2* expression from exomic and transcriptomic sequence data, respectively, were calculated for each tumor type. These median values were chosen to minimize the effect of uncontrolled variables and extreme results for individual cancer types. Although the mutation load varied among the 33 cancer types in TCGA, we found that the median value for the C-to-T mutation load correlated positively with expression of *A3B* (Spearman’s r = 0.7706, *P* < 0.0001) and of *MYBL2* (Spearman’s r = 0.5964, P = 0.0002) (Fig. 5B,C). This result was consistent with that of Burns *et al*.^8^ and supported the idea that B-Myb triggers *A3B* expression, leading to somatic mutation in multiple cancers. Furthermore, we assessed gene expression in the 16 cancer types in which both *A3B* and *MYBL2* were markedly upregulated (*P* < 0.001). As expected, *A3B* expression correlated with that of *MYBL2* in 15 of the 16 cancer types (Fig. 5D; see also Supplementary Table S2). Although this finding showed that the correlation between *A3B* and *MYBL2* varied, it was apparent that the higher correlations were more prevalent in the cancer types that had a relatively lower median mutation load (*P* = 0.0055, Spearman’s r = −0.6588; Fig. 5D), i.e., *A3B* expression is regulated by B-Myb. Among the cancer types having a higher median mutation load, factors acting in addition to B-Myb may contribute to increasing *A3B* expression.

### EGFR Inhibition Attenuates *MYBL2* and *A3B* Expression

**Figure 6:**
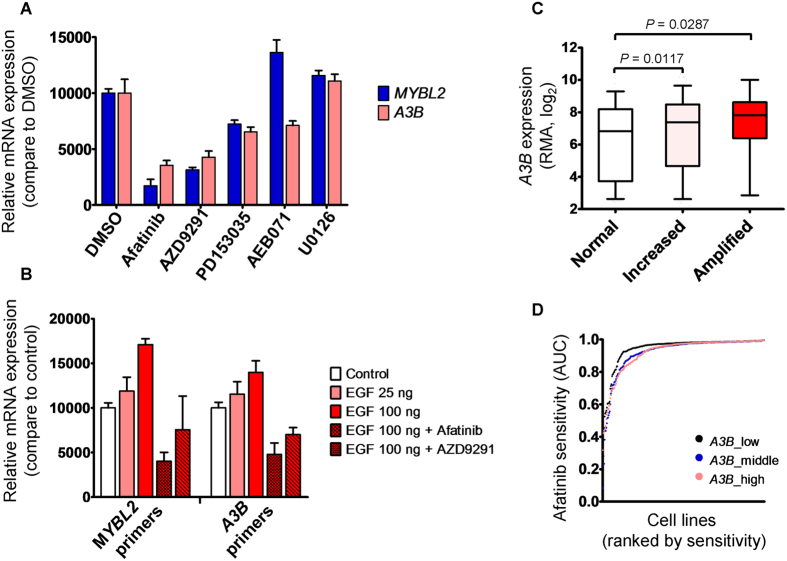
Inhibition of EGFR activity attenuates *MYBL2* and *A3B* expression. (**A**) Histogram showing each *MYBL2* and *A3B* expression value and 95% confidence interval relative to *TBP* expression. T47D cells that had been treated with 10 μM afatinib, 10 μM AZD9291, 2 μM PD153035, 10 μM AEB071, or 1 μM U0126 for 24 h were subjected to RNA extraction for gene expression analysis by quantitative RT-PCR. Asterisks indicate that *MYBL2* or *A3B* expression in those treatments differed significantly from that of the DMSO treatment (control). (**B**) As in (**A**), T47D cells that had been treated with 10 μM afatinib, 10 μM AZD9291, or DMSO for 30 min were incubated with 25 or 100 ng/ml EGF for 24 h before RNA extraction. (**C**) Histogram showing the median value and 5th–95th percentile range of the *A3B* RMA values from 829 GDSC cell lines. Statistical significance between two groups was determined with the Student’s *t*-test (*P* for trend = 0.0264). *EGFR* copy number status was defined as follows: normal, copies ≤2 (248 cell lines); increased, 3≤ copies ≤7 (560 cell lines); amplified, copies ≥8 (21 cell lines). (**D**) Dot plot showing afatinib sensitivity, presented by AUC values, in different groups based on low (black circle), middle (blue circle), and high (pink circle) third ranks of *A3B* RMA values. Cell lines listed on the *x* axis were ranked according to afatinib sensitivity. The AUC values in the low third group were statistically different from those in the middle and high third groups at *P* < 0.0001 and *P* = 0.0023, respectively (Mann–Whitney *U*-test).

The A3B deaminase activity promotes DNA hypermutation in multiple cancers and portends a poor prognosis, and therefore inhibition of A3B activity could potentially improve therapy. Although this idea is reasonable, research will be required to identify a viable drug candidates. Alternatively, *A3B* expression could be downregulated at the transcription level. Here, noting that *MYBL2* is a downstream target of EGFR^28^, we focused on regulating *A3B* expression using EGFR inhibitors. To gain insight into whether EGFR regulates *A3B* expression as well as *MYBL2*, T47D cells were treated with three EGFR inhibitors. As shown in Fig. 6A, three of these inhibitors could repress both *MYBL2* and *A3B* expression compared with the potent PKC inhibitor, AEB071. In contrast, adding EGF promoted *MYBL2* and *A3B* expression in T47D cells, whereas induction of these genes could be abolished by EGFR inhibitors such as afatinib and AZD9291 (Fig. 6B). In particular, although AEB071 downregulated *A3B* expression, this was not the case for *MYBL2*, indicating that at least two parallel signal pathways act synergistically to regulate *A3B* expression in human cells (Fig. 6B). The *EGFR* sequence is frequently altered in multiple cancers, and if B-Myb indeed regulates *A3B* expression and somatic mutations are mediated via EGFR activity, this could favor the clinical tack of inhibiting EGFR signaling in cancer therapeutics. To address this possibility, we noted that the Genomics of Drug Sensitivity in Cancer (GDSC) database has established interaction datasets for individual genes and drugs for more than 1000 human cancer cell lines^29^. Consequently, from 852 cell lines, we obtained both afatinib sensitivity results, as represented by the area under curve (AUC) value, and normalized gene expression values, and particularly *EGFR* copy numbers were evaluated for 829 of these cell lines. Accordingly, we found that *EGFR*-amplified cell lines were relatively more sensitive to afatinib, resulting in lower AUC values (Supplementary Fig. S4A). Analysis of this GDSC dataset also revealed that *EGFR*-amplified cells tend to have elevated *A3B* expression (Fig. 6C), which is consistent with our finding that EGF stimulated *A3B* expression in T47D cells. Moreover, we found these cell lines which exhibited upregulated *EGFR* or *A3B* expression were markedly sensitive to afatinib (Fig. 6D and Supplementary Fig. S4B). This is of special interest because no A3B-specific antagonist has been identified, and our results suggest a rational pathway for preventing *A3B* expression in human cancer cells.

## Discussion

Recent advances in sequencing technology have provided platforms that allow hundreds of thousands of variants in cancer genomes to be comprehensively detected, thus providing a basis for identifying driver mutations without prior knowledge of function^30^,^31^. Among distinct mutational signatures, some are present in many cancer types, and, notably, a signature that is enriched for the generation of C-to-T mutations was attributed to overexpression of *A3B*^8^,^12^. In the present study, we used two parallel approaches to determine the mechanism for *A3B* upregulation in cancer. Using a cell-based study, we scanned the active *A3B* promoter region, which narrowed the list of potential candidates that regulate this gene, and only the transcription factor B-Myb could induce *A3B* expression and promote cytosine editing in DNA of breast cancer cells. Additionally, the DNA hyerpmutation triggered by B-Myb is less efficient than by A3B, suggesting that B-myb acts upstream of A3B and induces *A3B* expression than directly catalyzes C residues deamination in breast cancer cells. In a complementary approach, querying TCGA dataset allowed us to determine clinically meaningful factors, and thus we found *MYBL2* expression was also upregulated and correlated with *A3B* expression in breast and multiple cancer types, implying that B-Myb might act as a general transcription factor for *A3B* induction, and *MYBL2* upregulation could explain how *A3B* is upregulated in multiple human cancers. Besides our finding, it has been demonstrated that HPV infection or activation of the PKC/NF-κB pathway upregulates *A3B* expression in breast cancer^19^,^22^. Our finding might be more clinically relevant because *MYBL2* has been used as aggressive prognostic marker for breast cancer^24^, and its expression, reflected by a functional SNP rs619289, significantly modifies breast cancer risk and predicts a poor outcome. We found that *MYBL2* overexpression is also associated with elevated somatic mutation load in breast cancer cells and tumors as evidenced by the fact that B-Myb is a critical factor for A3B-induced DNA mutation, although we cannot rule out the possibility that B-Myb regulates other DNA-modifying enzymes. Further comprehensive regulatory network analysis will improve insight into B-Myb–dependent cancer mutagenesis.

In our study, we evaluated the association between an *MYBL2*-adjacent SNP and disease-free survival in breast cancer patients. The rs619289 polymorphism is located within 1.1 kb upstream of the *MYBL2* TSS—theoretically within the promoter region. Given the proximity of this SNP to the TSS, our findings support that rs619289 might be a *cis*-regulated eQTL SNP for MYBL2 and a *trans*-regulated SNP for A3B. To our knowledge, it is unclear which factors can directly bind the region near rs619289, and no adjacent linkage disequilibrium variant could enable us to clarify how rs619289 might regulate *MYBL2*. Nonetheless, our present data and previous results conclusively show that individuals who carry the T allele of rs619289 have significantly greater *MYBL2* expression and, consequently, elevated breast cancer risk and poorer outcome^26^.

Elevated *A3B* expression is a known indicator of poor prognosis for breast cancer and other cancers, and therefore targeting A3B activity would be a rational therapeutic strategy. To our knowledge, no candidate compound has been identified that directly targets B-Myb or A3B. Alternatively, the pan-PKC inhibitor AEB071 has been tested, and it has been proposed to inhibit *A3B* expression and prevent metastasis of primary tumors^22^. Because EGFR acts as transcription factor that regulates *MYBL2*^28^, we achieved *A3B* suppression using three different EGFR inhibitors (Fig. 6A), suggesting their potential use as alternatives to AEB071. Therefore, we analyzed cytotoxic response data from the GDSC database and found a correlation between *A3B* expression and sensitivity of cancer cells to afatinib. Notably, EGFR action here presumably depends on its nuclear translocation^28^, whereas the precise role of the inhibitors we used in interfering with EGFR-directed transcriptional activity was not thoroughly evaluated. Rather, our study demonstrates that afatinib is efficient at targeting *A3B*, and thus afatinib may be effective against *A3B*-overexpressing tumors. This result was also consistent with the recent finding that inhibition of EGFR and ERBB2 by afatinib and lapatinib were able to repress replication stress-induced *A3B* expression in breast cancer cells^32^.

*EGFR* mutation and gene amplification are widespread in the majority of TCGA-investigated cancer types^7^,^33^,^34^, and thus it might be fruitful to investigate the involvement of the B-Myb–A3B pathway activation in these cancers. Alterations in the conformational state of EGFR, e.g., by phosphorylation, lead to changes in EGFR-mediated transcriptional regulation as well as many aspects of downstream signal transduction, including that mediated by the RAS-MAPK, PI3K-AKT, and JAK-STAT pathways^35^. Considering the heterogenic effects *EGFR* mutations, we opted to restrict our evaluation to changes in *EGFR* gene transcription that occur because of *EGFR* amplification in cancer cell lines. Recently, cancers that involve *EGFR* mutation have become more troublesome to treat, and hence much effort has been invested to overcome resistance to current drugs^36^. In summary, our work describes a novel mechanism by which B-Myb promotes *A3B* expression linked to *EGFR* amplification (and corresponding changes in EGFR signaling) in numerous cancer types. Our results improve our understanding of the involvement of EGFR signaling in cancer, toward the goal of helping prevent cancer-related mutagenesis and drug resistance.

## Methods

### Cell Lines and Reagents

Human breast cancer MCF7 cells were cultured as described^37^. T47D and Hs578T cells were obtained from the Bioresource Collection and Research Center (Hsinchu, Taiwan) and cultured in RPMI 1640 medium or DME medium (Sigma-Aldrich) supplemented with 10 μg/ml insulin (Thermo Fisher Scientific). All cells were maintained in medium supplemented with 10% fetal bovine serum (Thermo Fisher Scientific). Plasmid or siRNA transfection for all cells was performed using Lipofectamine 2000 as described^37^. To induce EGFR signaling, EGF (Peprotech) was added to serum-free T47D culture medium with subsequent incubation for 24 h. Afatinib, AZD9291, PD153035, and AEB071 were purchased from Selleckchem, and the MEK inhibitor U0126 was obtained as described^38^.

### siRNAs and Plasmids

The control siRNA has been described^37^, and *MYBL2* siRNA #1 (HSS106825), #2 (HSS106826), and #3 (HSS106827) were obtained from Thermo Fisher Scientific. Individual full-length cDNAs encoding each of *ETS2* (NM_005239.5), *NR1H4* (NM_005123.3), *JUN* (NM_002228), *MAZ* (NM_002383.3), *MYBL2* (NM_002466.3), *NFYA* (NM_002505.4), and *APOBEC3B* (NM_004900.4) were amplified from human breast cancer cells and cloned into Flag-tagged pcDNA3 vector for mammalian expression^38^. Owing to sequence instability^39^, we cloned the full-length *A3B* sequence containing a 206-bp insertion within intron 3. The full-length *UGI* coding sequence from bacteriophage PBS2 was synthesized by Genomics Company (Taipei, Taiwan) and then subcloned into Flag-tagged pcDNA3. The full-length *A3B* promoter (−1005 to +65) and truncated sequence were amplified from MCF7 genomic DNA and then cloned into pGL3. Supplementary Table S3 lists all primer pairs used for plasmid construction.

### Quantitative PCR

mRNAs were extracted and then quantified via quantitative reverse-transcription PCR (RT-PCR) as described^38^. The results for each gene were normalized to those for *TBP* and measured using the comparative CT method. The mean and 95% confidence interval for each gene in each treatment (three experimental replicates) were calculated using the Student’s *t*-test, and three independent assays were performed. The primer pairs used in this study were 5′-CTATGGTCGGAGCTACACTTGG-3′ and 5′-GGAAACACTTGTAAGCAGGCAG-3′ for *A3B*^40^, 5′-CAGAGCCCTTGGAGGAATT-3′ and 5′-CAGGCTCGTTTCTGGTGG-3′ for *MYBL2*^41^, and 5′-CACGAACCACGGCACTGATT-3′ and 5′-TCACATCACAGCTCCCCACC-3′ for *TBP*.

To identify the DNA region to which B-Myb binds, ChIP assay was preformed using the EZ-Magna ChIP^TM^ G kit (Merck-Millipore). The DNA precipitated with anti-B-Myb (ABD32, from Merck-Millipore) was eluted from immunoprecipitation beads with 40 μl water, and 6 μl of the eluted DNA was used for quantification in 15-μl quantitative PCR reactions. Supplementary Table S3 lists the primer pairs used for ChIP–coupled PCR.

### Luciferase Reporter Assay

The *A3B* promoter–derived pGL3 constructs were co-transfected with various transcription factor expression vectors or siRNAs into 50,000 cells in 24-well plates, and pRL-tk, which encodes renilla luciferase, was also co-transfected as an internal control as described^38^. After 48 h, cell extracts were prepared and luciferase activities measured using the Dual-Luciferase Reporter Assay System (Promega). Promoter activity was determined by calculating relative luciferase activity between firefly and renilla luciferase. The mean and 95% confidence interval for each group was calculated using the Student’s *t*-test from at least three independent wells in each experiment, and at least two independent experiments were performed.

### 3D-PCR and Clone Sequencing

The foreign DNA editing assay was carried out as described^25^. Briefly, we transfected pGL3-basic, pcDNA3-*UGI*, and *A3B* or *MYBL2* expression vectors into MCF7 cells. After 48 h transfection, total DNA was extracted using the Purelink Genomic DNA mini kit (Thermo Fisher Scientific), and we amplified the luciferase gene using 50 ng DNA as template. The first-round PCR was performed using Taq polymerase (Bioman), and the reaction parameters were 30 s at 94 °C, followed by 20 cycles of 30 s at 94 °C, 30 s at 52 °C, and 60 s at 68 °C, with a final 10 min at 72 °C. We used 0.2 μl of the first-round PCR products as template for nested PCR using KOD Hot Start DNA polymerase (Merck-Millipore), and the reaction parameters were 2 min at 95 °C, followed by 25 cycles of 20 s at 84–86.5 °C, 10 s at 50 °C, and 10 s at 70 °C, with a final 2 min at 70 °C. Supplementary Table S3 lists the primer pairs. PCR products were visualized with ethidium bromide after electrophoresis on a 1% agarose gel. We recovered PCR amplicons derived at 85 °C from agarose gels using the Qiaquick Gel Extraction kit (Qiagen), cloned them using the CloneJET PCR Cloning kit (Thermo Fisher Scientific), and then picked 10 colonies for sequence analysis to determine DNA editing sites.

### Breast Cancer Patients, Genotyping, Clinical Features, and Follow-up

The breast cancer TissueScan cDNA array (BCRT101) containing 48 normal and breast tumor samples was obtained from Origene. Only 46 of the 48 samples for *A3B* and *MYBL2* expression could be analyzed by respective primer pairs using quantitative RT-PCR because 2 samples could not be quantified. Genotyping data for rs619289 in 717 breast cancer patients were used to assess any association between genotypic polymorphisms of *MYBL2* and disease-free survival, as described^38^. Genotyping of blood specimens was achieved using iPLEX (Sequenom, Hamburg, Germany), and details of the clinical and pathological features were obtained from the tumor registries of our hospitals, the quality of which is well recognized^38^.

### Public Dataset Analysis

#### TCGA Portal

For somatic mutations analysis, level 2 exome sequence results for 33 cancer types were retrieved from TCGA Data Matrix on 31 January 2016. We removed those entries for insertion-deletion, adjacent multiple mutations, and mitochondrial DNA mutations, and the remaining mutations were then divided into six nucleotide-change categories to obtain C-to-T or G-to-A specific mutations. RNA-sequence expression values, including 33 cancer types, were retrieved from TCGA Data Matrix on 15 February 2016 and normalized by *TBP* expression as described^37^. Male breast cancer samples were removed before. Differential gene expression between normal and tumor samples of 21 cancer types was determined with the Mann–Whitney *U*-test. Supplementary Tables S4–S6 present the summary of TCGA RNA sequence expression and mutation results for each tumor type.

#### GTEx Portal

The rs619289 genotypes associated with *MYBL2* or *A3B* expression in whole-blood samples were retrieved from GTEx Analysis Release V6p (www.gtexportal.org), and statistical significance was determined using the website’s algorithm^27^.

#### GDSC Database

To explore the relationship between gene expression and afatinib sensitivity, the files containing Affymetrix Human Genome U219 array robust multi-array average (RMA)-normalized data (E-MTAB-3610), Affymetrix SNP6 processed copy number status, and processed AUC value for afatinib (drug id: 1032) treatment of 852 cell lines were retrieved from the GDSC database Release 6 (www.cancerrxgene.org) on 10 July 2016^29^. Statistical analysis was performed with Prism v5 (GraphPad Software) using the unpaired two-tailed *t*-test.

## Additional Information

**How to cite this article:** Chou, W.-C. *et al*. B-Myb Induces *APOBEC3B*Expression Leading to Somatic Mutation in Multiple Cancers. *Sci. Rep.* **7**, 44089; doi: 10.1038/srep44089 (2017).

**Publisher's note:** Springer Nature remains neutral with regard to jurisdictional claims in published maps and institutional affiliations.

## Supplementary Material

Supplementary Information
