# Supplementary material for: B-Myb Induces APOBEC3B Expression Leading to Somatic Mutation in Multiple Cancers
Source: Sci Rep. 2017 Mar 9;7:44089. doi: 10.1038/srep44089 (PMC5343453; doi:10.1038/srep44089)
Supplement: Supplementary Information [file srep44089-s1.pdf]

# B-Myb Induces *APOBEC3B* Expression Leading to Somatic Mutation in Multiple Cancers

Wen-Cheng Chou[1], Wei-Ting Chen[1], Chia-Ni Hsiung[1], Ling-Yueh Hu[1], Jyh-Cherng Yu[2], Huan-Ming Hsu[2], and Chen-Yang Shen[1,3,4,*]

[1]Institute of Biomedical Sciences, Academia Sinica, Taipei, Taiwan

[2]Department of Surgery, Tri-Service General Hospital, Taipei, Taiwan

[3]Taiwan Biobank, Academia Sinica, Taipei, Taiwan

[4]College of Public Health, China Medical University, Taichung, Taiwan

*Corresponding author.
Tel: +886-2-27899036; Fax: +886-2-27823047; E-mail:
bmcys@ibms.sinica.edu.tw

# Supplementary Information

## Supplementary Figure Legends

**Figure S1.** *A3B* and *MYBL2* expression levels correlate with somatic mutation counts in breast cancer. Each data point represents the total somatic mutation count for one sample in TCGA breast cancer dataset. Samples were grouped based on *A3B* or *MYBL2* expression level, and each horizontal bar indicates the median value. Asterisks indicate statistical significance between two groups (Mann–Whitney *U*-test), as indicated.

**Figure S2.** Expression of *APOBEC3* genes in the GTEx Portal. Correlations between the expression of *APOBEC3* genes and SNP rs619289 in whole-blood samples were queried from the GTEx Portal.

**Figure S3.** *A3B* is upregulated in multiple cancer types. Each data point represents relative *A3B* expression of one normal or tumor sample, and 21 cancer types (*x* axis) were queried from TCGA RNA sequence database. Blue and pink horizontal bars indicate the median expression for each normal and tumor cancer type, respectively. Asterisks indicate statistically significant *A3B* upregulation in the tumor type relative to the corresponding normal tissues.

**Figure S4.** Elevated *EGFR* expression is sensitive to afatinib treatment. (**A**) Histogram showing the median value and 5th–95th percentile range of afatinib AUC values queried from the GDSC database. *EGFR*-amplified cell lines were significantly sensitive to afatinib relative to non-amplified cell lines ($P = 0.0011$; Mann–Whitney *U*-test). (**B**) A dot plot shows the afatinib AUC values in

different groups based on low, middle, and high third ranks of *EGFR* RMA values in black, blue, and pink, respectively. The cell lines noted on the *x* axis were ranked according to AUC values, and the AUC values in the low third group were statistically different from those in the middle and high third groups at $P < 0.0001$ (Mann–Whitney *U*-test).

**Supplementary Figures**

# Figure S1

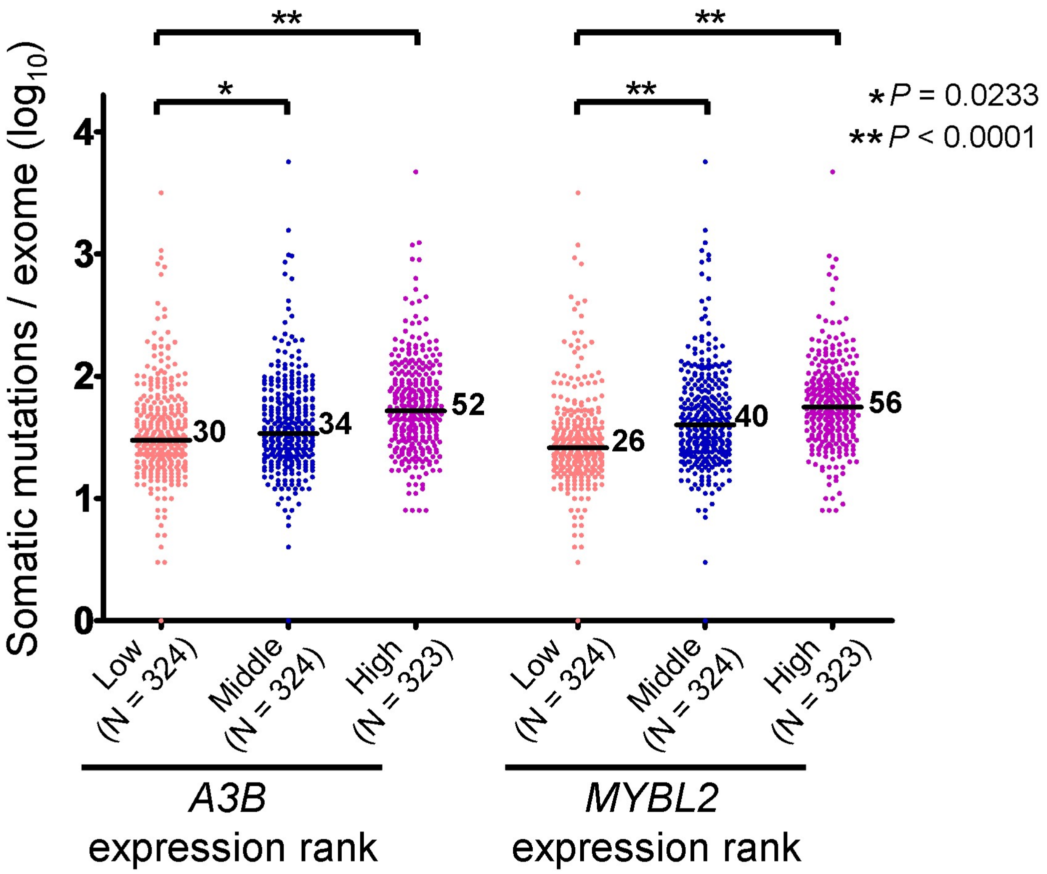

# Figure S2

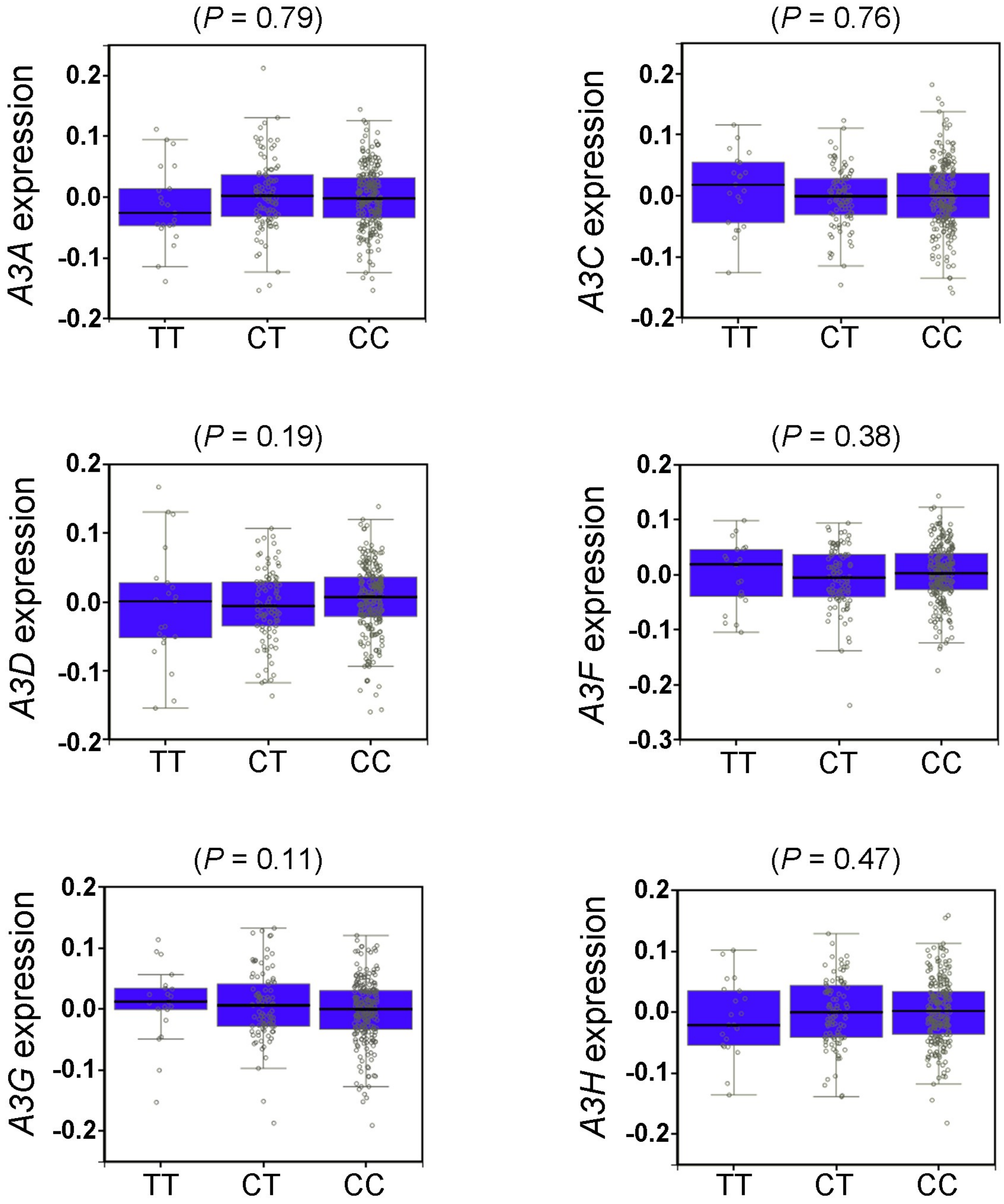
(P = 0.79)
A3A expression
0.2
0.1
0.0
-0.1
-0.2
TT
CT
CC
(P = 0.76)
A3C expression
0.2
0.1
0.0
-0.1
-0.2
TT
CT
CC
(P = 0.19)
A3D expression
0.2
0.1
0.0
-0.1
-0.2
TT
CT
CC
(P = 0.38)
A3F expression
0.2
0.1
0.0
-0.1
-0.2
-0.3
TT
CT
CC
(P = 0.11)
A3G expression
0.2
0.1
0.0
-0.1
-0.2
TT
CT
CC
(P = 0.47)
A3H expression
0.2
0.1
0.0
-0.1
-0.2
TT
CT
CC

# Figure S3

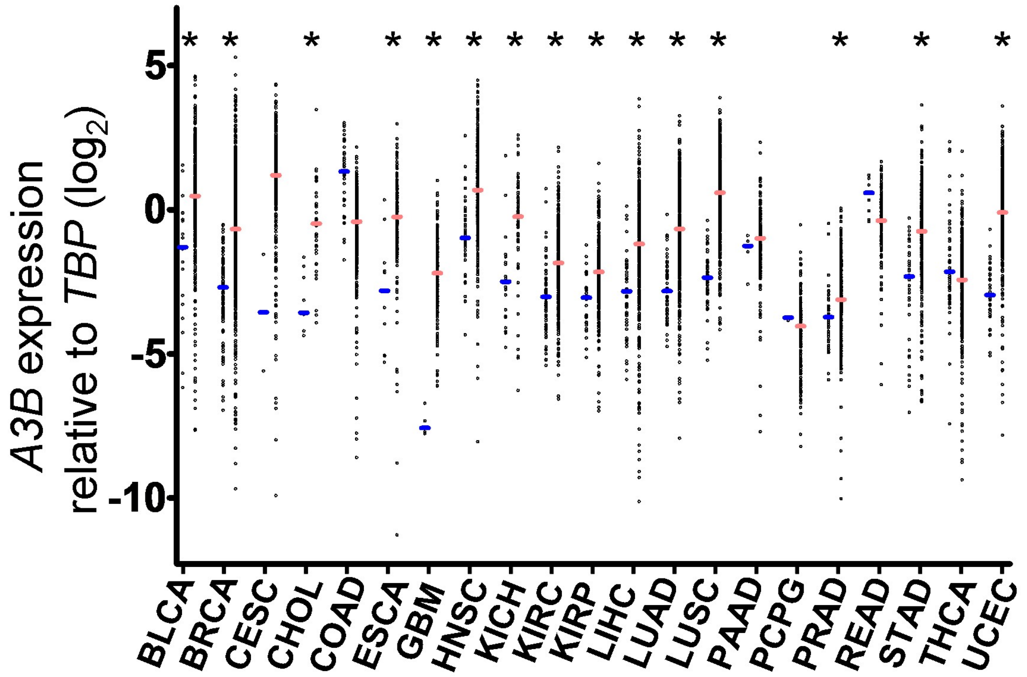

A3B expression relative to TBP (log2)
5
0
-5
-10
BLCA
BRCA
CESC
CHOL
COAD
ESCA
GBM
HNSC
KICH
KIRC
KIRP
LIHC
LUAD
LUSC
PAAD
PCPG
PRAD
READ
STAD
THCA
UCEC

## Figure S4A

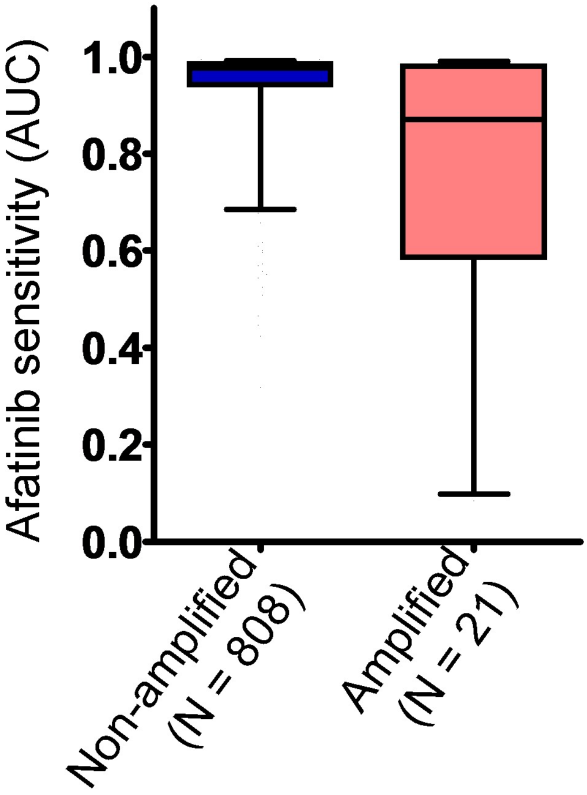


## Figure S4B

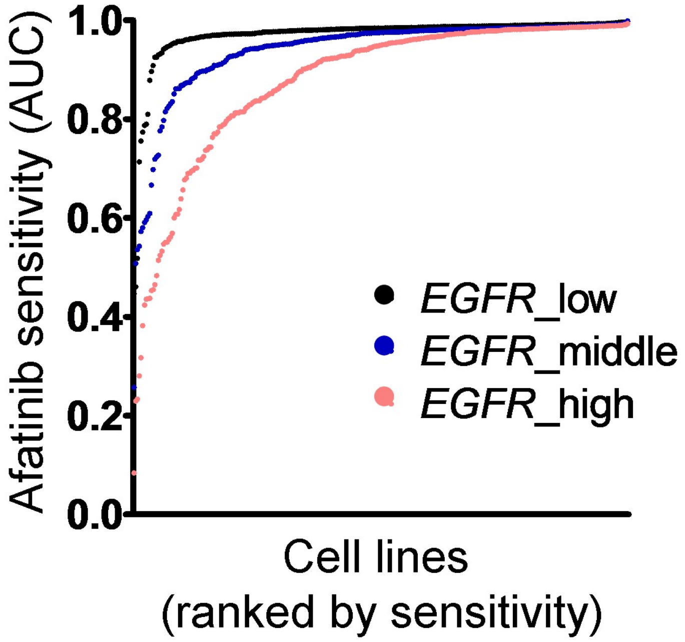

## Supplementary Tables

**Table S1.** Factors associated with disease-free survival of breast cancer (≥ stage II) in the Cox proportional hazard model. CI, confidence interval.

| Variable | Patients n. (%) | Event n. (%) | Hazard ratio (95%CI) | *P* value |
|---|---|---|---|---|
| rs619289 | | | | |
| CC | 487 | 104 (21.4) | | |
| CT | 215 | 63 (29.3) | | |
| TT | 15 | 5 (33.3) | 1.32 (1.01–1.73) | 0.04 |
| Stage | | | | |
| II | 472 | 72 (15.3) | | |
| III | 198 | 61 (30.8) | | |
| IV | 47 | 39 (83.0) | 4.54 (3.53–5.83) | <0.001 |
| Hormone therapy | | | | |
| No | 216 | 65 (30.1) | | |
| Yes | 411 | 97 (23.6) | 0.5 5(0.39–0.76) | 0.0003 |

**Table S2.** Correlation between *APOBEC3B* and *MYBL2* expression in 16 TCGA cancer types.

| Tumor Type | TCGA ID | Mutation (median) | Sample (n) | Spearman's r | *P* value |
|---|---|---|---|---|---|
| Prostate adenocarcinoma | PRAD | 9 | 497 | 0.6087 | < 0.0001 |
| Breast invasive carcinoma | BRCA | 18 | 1090 | 0.5690 | < 0.0001 |
| Kidney renal clear cell carcinoma | KIRC | 19 | 534 | 0.6713 | < 0.0001 |
| Kidney renal papillary cell carcinoma | KIRP | 26 | 291 | 0.5780 | < 0.0001 |
| Kidney Chromophobe | KICH | 33 | 66 | 0.3034 | 0.0133 |
| Liver hepatocellular carcinoma | LIHC | 35 | 374 | 0.2931 | < 0.0001 |
| Uterine Corpus Endometrial Carcinoma | UCEC | 38.5 | 546 | 0.3966 | < 0.0001 |
| Glioblastoma multiforme | GBM | 43 | 167 | 0.3288 | < 0.0001 |
| Cholangiocarcinoma | CHOL | 59 | 36 | 0.1393 | 0.4179 |
| Lung adenocarcinoma | LUAD | 60 | 517 | 0.4139 | < 0.0001 |
| Head and Neck squamous cell carcinoma | HNSC | 63 | 522 | 0.3246 | < 0.0001 |
| Cervical squamous cell carcinoma and endocervical adenocarcinoma | CESC | 69 | 306 | 0.2951 | < 0.0001 |
| Esophageal carcinoma | ESCA | 70.5 | 185 | 0.2629 | 0.0003 |
| Stomach adenocarcinoma | STAD | 72 | 414 | 0.2533 | < 0.0001 |
| Lung squamous cell carcinoma | LUSC | 86.5 | 501 | 0.2238 | < 0.0001 |
| Bladder Urothelial Carcinoma | BLCA | 105 | 408 | 0.3686 | < 0.0001 |

**Table S3.** Primer sequences used in the study.

| Target gene | Nucleotide Sequence 5'→3' | Forward/ Reverse |
|---|---|---|
| ETS2 cds | GATGAATTCATGAATGATTTCGGAATCAA | F |
| | GATCTCGAGTCAGTCCTCCGTGTCGGG | R |
| NR1H4 cds | AAACGGCCGATGGGATCAAAAATGAATCTCATTGAACATT C | F |
| | CCCCTCGAGTCCCCATCACTGCACGTCCCAGATTTCAC | R |
| JUN cds | GACGAATTCATGACTGCAAAGATGGAAACGACCTTC | F |
| | GGTCTCGAGTCAAAATGTTTGCAACTGCTGCGTTAG | R |
| MAZ cds | GCCGAATTCATGTTCCCGGTGTTTCCTTGCACGCTG | F |
| | AACCTCGAGCTCACCAGGGTTGGGAGGGAAGTGGC | R |
| MYBL2 cds | ATACGGCCGATGTCTCGGCGGACGCGCTG | F |
| | TAGGTCGACCAGGACAAGATGAGGGTCCG | R |
| NFYA cds | GATGAATTCACCATGGAGCAGTATACAGC | F |
| | GATCTCGAGTTAGGACACTCGGATGATC | R |
| APOBEC3B cds | AAGAAGCTTAACATGAATCCACAGATCAGAAATCCG | F |
| | TAGCGGTACCGTTTCCCTGATTCTGGAGAATG | R |
| APOBEC3B intron3 | GAGTCAGGCAGGAGCCCGCGTGAAGATC | F |
| | TGTACACAAAGTTTTCCCAGCAGTATGCAAATT | R |
| A3B promoter (-1005 ~ +65) | AAAGGTACCAGAAAAGAAAACTCACCTTCC | F |
| | ACCAGATCTGTGGATTCATGTTCAGCCTC | R |
| A3B promoter (-1005 ~ -93) | AAAGGTACCAGAAAAGAAAACTCACCTTCC | F |
| | CCCAGATCTGCTCAGGCATTGGTGTGG | R |
| A3B promoter (-114 ~ +65) | CCCGGTACCTCCCACACCAATGCCTGAGC | F |
| | ACCAGATCTGTGGATTCATGTTCAGCCTC | R |
| A3B promoter (-114 ~ +17) | CCCGGTACCTCCCACACCAATGCCTGAGC | F |
| | TCCAGATCTTTTTTTGAAGCTCTGTGG | R |
| A3B promoter (-19 ~ +65) | CAAGGTACCTGTAAGCAGGAAGTGAAAC | F |
| | ACCAGATCTGTGGATTCATGTTCAGCCTC | R |
| A3B promoter (for ChIP) | GGCCCTGGGAGGTCACTT | F |
| | CTGCTTACAGCGTCCTTGCA | R |
| Luc+ (First round) | CCAACCCTATTCTCCTTCTTC | F |
| | AAACCTCCCACACCTCCCCC | R |
| Luc+ (Nested) | CAGCTATTCTGATTACACCC | F |
| | TAATCCACGATCTCTTTTTCC | R |

**Table S4.** Gene expression summary for TCGA tumor samples in this study.

| Tumor Type | TCGA ID | Samples (n) | APOBEC3B Range | APOBEC3B Median | MYBL2 Range | MYBL2 Median |
|---|---|---|---|---|---|---|
| Adrenocortical carcinoma | ACC | 79 | 0.011–12 | 1.0 | 0.011–7.7 | 0.55 |
| Bladder Urothelial Carcinoma | BLCA | 408 | 0–25 | 1.4 | 0.036–62 | 6.7 |
| Breast invasive carcinoma | BRCA | 1090 | 0–39 | 0.63 | 0.0055–49 | 2.8 |
| Cervical squamous cell carcinoma and endocervical adenocarcinoma | CESC | 306 | 0.0010–20 | 2.3 | 2.3–85 | 8.4 |
| Cholangiocarcinoma | CHOL | 36 | 0.066–11 | 0.71 | 0.23–9.3 | 2.2 |
| Colon adenocarcinoma | COAD | 460 | 0.0026–4.5 | 0.75 | 0.49–39 | 7.1 |
| Lymphoid Neoplasm Diffuse Large B-cell Lymphoma | DLBC | 48 | 0–9.4 | 0.59 | 5.8–78 | 24 |
| Esophageal carcinoma | ESCA | 185 | 0.00040–7.9 | 0.84 | 1.4–56 | 7.9 |
| Glioblastoma multiforme | GBM | 167 | 0.014–2.0 | 0.22 | 0.064–10 | 2.6 |
| Head and Neck squamous cell carcinoma | HNSC | 522 | 0.0038–22 | 1.6 | 1.2–70 | 6.0 |
| Kidney Chromophobe | KICH | 66 | 0.013–6.0 | 0.84 | 0.0059–11 | 0.12 |
| Kidney renal clear cell carcinoma | KIRC | 534 | 0–4.5 | 0.28 | 0.0064–10 | 0.36 |
| Kidney renal papillary cell carcinoma | KIRP | 291 | 0.0079–3.0 | 0.22 | 0.0050–9.5 | 0.29 |
| Acute Myeloid Leukemia | LAML | 173 | 0.018–2.8 | 0.46 | 0.14–11 | 2.2 |
| Brain Lower Grade Glioma | LGG | 530 | 0–1.3 | 0.054 | 0–16 | 0.20 |
| Liver hepatocellular carcinoma | LIHC | 374 | 0–14 | 0.44 | 0.04–60 | 1.7 |
| Lung adenocarcinoma | LUAD | 517 | 0–9.6 | 0.63 | 0.098–32 | 3.2 |
| Lung squamous cell carcinoma | LUSC | 501 | 0.056–15 | 1.5 | 0.037–35 | 6.0 |
| Mesothelioma | MESO | 87 | 0.033–2.5 | 0.46 | 0.13–13 | 2.1 |
| Ovarian serous cystadenocarcinoma | OV | 308 | 0.0015–8.6 | 0.51 | 0.10–36 | 5.4 |
| Pancreatic adenocarcinoma | PAAD | 179 | 0.0048–5.1 | 0.50 | 0.012–16 | 1.8 |
| Pheochromocytoma and Paraganglioma | PCPG | 184 | 0–0.69 | 0.06 | 0.0053–2.1 | 0.055 |
| Prostate adenocarcinoma | PRAD | 497 | 0–1.0 | 0.11 | 0.016–22 | 0.35 |
| Rectum adenocarcinoma | READ | 167 | 0.015–3.2 | 0.77 | 0.48–114 | 8.4 |
| Sarcoma | SARC | 263 | 0.0014–5.5 | 0.56 | 0.11–28 | 2.7 |
| Skin Cutaneous Melanoma | SKCM | 472 | 0.0011–10 | 0.99 | 0.080–93 | 4.1 |
| Stomach adenocarcinoma | STAD | 414 | 0.0099–12 | 0.59 | 0.16–45 | 7.0 |
| Testicular Germ Cell Tumors | TGCT | 156 | 0.0073–1.8 | 0.15 | 0.46–162 | 37 |
| Thyroid carcinoma | THCA | 513 | 0–4.1 | 0.18 | 0.0013–6.1 | 0.15 |
| Thymoma | THYM | 120 | 0.011–8.6 | 0.14 | 0.063–51 | 6.1 |
| Uterine Corpus Endometrial Carcinoma | UCEC | 546 | 0–12 | 0.93 | 0.44–90 | 6.7 |
| Uterine Carcinosarcoma | UCS | 57 | 0.0038–3.7 | 0.52 | 1.8–25 | 6.2 |
| Uveal Melanoma | UVM | 80 | 0.0076–1.2 | 0.24 | 0.0030–7.2 | 0.56 |

**Table S5.** Gene expression summary for TCGA normal samples in this study.

| Tumor Type | TCGA ID | Samples (n) | APOBEC3B Range | APOBEC3B Median | MYBL2 Range | MYBL2 Median |
|---|---|---|---|---|---|---|
| Adrenocortical carcinoma | ACC | n.a. | | | | |
| Bladder Urothelial Carcinoma | BLCA | 19 | 0.014–2.9 | 0.41 | 0.0028–22 | 0.66 |
| Breast invasive carcinoma | BRCA | 112 | 0.0081–0.70 | 0.15 | 0.021–3.0 | 0.17 |
| Cervical squamous cell carcinoma and endocervical adenocarcinoma | CESC | 3 | 0.021–0.34 | 0.085 | 0.019–0.35 | 0.037 |
| Cholangiocarcinoma | CHOL | 9 | 0.049–0.32 | 0.084 | 0.024–0.17 | 0.044 |
| Colon adenocarcinoma | COAD | 41 | 0.30–8.1 | 2.5 | 0.27–5.0 | 2.1 |
| Lymphoid Neoplasm Diffuse Large B-cell Lymphoma | DLBC | n.a. | | | | |
| Esophageal carcinoma | ESCA | 11 | 0.026–1.3 | 0.14 | 0.012–6.8 | 0.48 |
| Glioblastoma multiforme | GBM | 5 | 0.0046–0.0095 | 0.0053 | 0.0016–0.014 | 0.011 |
| Head and Neck squamous cell carcinoma | HNSC | 44 | 0.049–5.9 | 0.51 | 0.082–3.9 | 1.9 |
| Kidney Chromophobe | KICH | 25 | 0.037–3.7 | 0.18 | 0.0013–0.66 | 0.018 |
| Kidney renal clear cell carcinoma | KIRC | 72 | 0.024–1.7 | 0.12 | 0–3.2 | 0.017 |
| Kidney renal papillary cell carcinoma | KIRP | 32 | 0.029–0.43 | 0.12 | 0.0012–0.24 | 0.016 |
| Acute Myeloid Leukemia | LAML | n.a. | | | | |
| Brain Lower Grade Glioma | LGG | n.a. | | | | |
| Liver hepatocellular carcinoma | LIHC | 50 | 0.017–1.1 | 0.14 | 0.017–0.87 | 0.090 |
| Lung adenocarcinoma | LUAD | 59 | 0.037–0.89 | 0.14 | 0.049–5.2 | 0.14 |
| Lung squamous cell carcinoma | LUSC | 51 | 0.027–0.77 | 0.19 | 0.013–2.0 | 0.21 |
| Mesothelioma | MESO | n.a. | | | | |
| Ovarian serous cystadenocarcinoma | OV | n.a. | | | | |
| Pancreatic adenocarcinoma | PAAD | 4 | 0.17–0.54 | 0.42 | 0.41–5.0 | 0.78 |
| Pheochromocytoma and Paraganglioma | PCPG | 3 | 0.070–0.076 | 0.074 | 0.038–0.070 | 0.041 |
| Prostate adenocarcinoma | PRAD | 52 | 0.017–0.72 | 0.076 | 0.0020–4.2 | 0.055 |
| Rectum adenocarcinoma | READ | 10 | 0.75–2.3 | 1.5 | 0.77–4.7 | 2.5 |
| Sarcoma | SARC | n.a. | | | | |
| Skin Cutaneous Melanoma | SKCM | n.a. | | | | |
| Stomach adenocarcinoma | STAD | 35 | 0.0076–0.82 | 0.20 | 0.0051–6.3 | 1.8 |
| Testicular Germ Cell Tumors | TGCT | n.a. | | | | |
| Thyroid carcinoma | THCA | 59 | 0.0058–5.1 | 0.23 | 0.0038–10 | 0.026 |
| Thymoma | THYM | n.a. | | | | |
| Uterine Corpus Endometrial Carcinoma | UCEC | 35 | 0.030–0.62 | 0.13 | 0.0037–3.7 | 0.063 |
| Uterine Carcinosarcoma | UCS | n.a. | | | | |
| Uveal Melanoma | UVM | n.a. | | | | |

**Table S6.** Summary of mutation statistics for 33 TCGA cancer types in this study.

| Tumor Type | TCGA ID | Samples (n) | Total mutations | | C-to-T mutations | |
|---|---|---|---|---|---|---|
| | | | Range | Median | Range | Median |
| Adrenocortical carcinoma | ACC | 90 | 54–2504 | 130 | 20–750 | 48.5 |
| Bladder Urothelial Carcinoma | BLCA | 130 | 16–1759 | 213 | 5–1243 | 105 |
| Breast invasive carcinoma | BRCA | 973 | 0–5678 | 38 | 0–2802 | 18 |
| Cervical squamous cell carcinoma and endocervical adenocarcinoma | CESC | 194 | 3–2720 | 122.5 | 0–1261 | 69 |
| Cholangiocarcinoma | CHOL | 35 | 95–1034 | 151 | 36–207 | 59 |
| Colon adenocarcinoma | COAD | 269 | 0–6980 | 122 | 0–3573 | 76 |
| Lymphoid Neoplasm Diffuse Large B-cell Lymphoma | DLBC | 48 | 24–4667 | 151.5 | 10–2539 | 80 |
| Esophageal carcinoma | ESCA | 184 | 8–1939 | 150 | 4–1318 | 70.5 |
| Glioblastoma multiforme | GBM | 282 | 4–267 | 70 | 1–240 | 43 |
| Head and Neck squamous cell carcinoma | HNSC | 279 | 3–1985 | 132 | 0–1715 | 63 |
| Kidney Chromophobe | KICH | 66 | 42–1156 | 78.5 | 14–943 | 33 |
| Kidney renal clear cell carcinoma | KIRC | 417 | 12–142 | 59 | 2–47 | 19 |
| Kidney renal papillary cell carcinoma | KIRP | 161 | 17–308 | 84 | 7–115 | 26 |
| Acute Myeloid Leukemia | LAML | 197 | 0–43 | 11 | 0–28 | 7 |
| Brain Lower Grade Glioma | LGG | 286 | 1–526 | 28 | 0–487 | 15 |
| Liver hepatocellular carcinoma | LIHC | 198 | 2–1696 | 108.5 | 1–170 | 35 |
| Lung adenocarcinoma | LUAD | 230 | 19–1601 | 197 | 11–366 | 60 |
| Lung squamous cell carcinoma | LUSC | 178 | 0–3910 | 296 | 0–3410 | 86.5 |
| Mesothelioma | MESO | 83 | 36–591 | 81 | 3–385 | 21 |
| Ovarian serous cystadenocarcinoma | OV | 463 | 1–204 | 51 | 0–67 | 20 |
| Pancreatic adenocarcinoma | PAAD | 150 | 3–20056 | 61.5 | 1–12236 | 40 |
| Pheochromocytoma and Paraganglioma | PCPG | 184 | 7–53 | 19.5 | 2–24 | 9 |
| Prostate adenocarcinoma | PRAD | 332 | 1–943 | 26 | 0–117 | 9 |
| Rectum adenocarcinoma | READ | 116 | 0–7504 | 93 | 0–3662 | 55.5 |
| Sarcoma | SARC | 259 | 7–2766 | 63 | 2–2391 | 26 |
| Skin Cutaneous Melanoma | SKCM | 341 | 8–48340 | 409 | 3–47003 | 345 |
| Stomach adenocarcinoma | STAD | 289 | 0–8300 | 152 | 0–3478 | 72 |
| Testicular Germ Cell Tumors | TGCT | 155 | 38–521 | 77 | 11–189 | 25 |
| Thyroid carcinoma | THCA | 405 | 2–95 | 15 | 0–78 | 7 |
| Thymoma | THYM | 123 | 6–682 | 29 | 0–491 | 11 |
| Uterine Corpus Endometrial Carcinoma | UCEC | 248 | 1–14692 | 67.5 | 0–9405 | 38.5 |
| Uterine Carcinosarcoma | UCS | 57 | 29–4490 | 56 | 14–1759 | 29 |
| Uveal Melanoma | UVM | 80 | 8–578 | 17 | 3–569 | 8.5 |
